# Highly efficient genome editing using CRISPR/Cas9 ribonucleoprotein in the marine oleaginous diatom *Fistulifera solaris*

**DOI:** 10.1038/s41598-026-49816-1

**Published:** 2026-04-24

**Authors:** Rein Yasui, Sawa Suzuki, Taiga Fujii, Yusuke Yabu, Yoshiaki Maeda, Satoshi Murata, Kosuke Kataoka, Tsuyoshi Tanaka

**Affiliations:** 1 Division of Biotechnology and Life Science, Institute of Engineering, Tokyo University of Agriculture and Technology, 2-24-16 Naka-cho, Koganei, 184-8588 Tokyo, Japan; 2 Faculty of Life and Environmental Sciences, University of Tsukuba, 1-1-1 Tennoudai, Tsukuba, 305-8572 Ibaraki Japan; 3 Comprehensive Research Organization, Waseda University, Tokyo, 162- 0041 Japan

**Keywords:** Biotechnology, Genetics, Molecular biology, Plant sciences

## Abstract

Microalgae are promising hosts for sustainable aviation fuel production due to their rapid growth and their advantage of not competing with agricultural crops. The oleaginous diatom *Fistulifera solaris* is notable for its exceptionally high lipid content. However, genetic engineering tools for this species remain limited, partly due to its allodiploid genome structure. Here, we report the successful development of a CRISPR/Cas9 genome editing method using ribonucleoprotein (RNP) complexes in *F. solaris*. We first targeted the adenine phosphoribosyl transferase (*apt*) genes as a selectable marker. Delivery of RNPs via particle bombardment to conserved regions of the two homoeologous *apt* genes resulted in 87% biallelic editing efficiency. Next, we demonstrated multiplex genome editing by co-targeting the *apt* genes and diadinoxanthin de-epoxidase (*dde*) genes as representative targets for improving valuable compound production in diatoms. Among 27 co-edited clones, 85% showed mutations in at least one *dde* homoeolog, and 63% exhibited biallelic mutations in both genes. This study demonstrates the applicability of RNP-mediated genome editing in diatoms. Furthermore, the success of this method suggests its broader applicability to non-model diatom species, providing a valuable tool for genome engineering in diatoms.

**Supplementary Information:**

The online version contains supplementary material available at 10.1038/s41598-026-49816-1.

## Introduction

Microalgae have attracted attention as a host for production of sustainable aviation fuel due to their rapid growth rates compared to plants, and the advantage of not competing with agricultural crops for food production^1,2^. However, commercial applications of microalgae, such as biofuels, remain limited, mainly due to the high production costs. Metabolic engineering through gene overexpression and knockdown represents a promising strategy to enhance the productivity of valuable compounds^3,4^.

Genome editing techniques using the clustered regularly interspaced short palindromic repeats (CRISPR)/Cas9 system have been widely used due to their low cost and simplicity^5^. The CRISPR/Cas9 system requires both the Cas9 protein and a single-guide RNA (sgRNA), which can be introduced into microalgal cells either through vector-based expression or as ribonucleoprotein (RNP) complexes. RNP-mediated gene editing has several advantages, including avoiding genomic integration and enabling transient expression, thus reducing off-target effects and lowering cytotoxicity^6^. To date, successful genome editing using RNPs has been reported in several microalgal species, including *Chlamydomonas reinhardtii*^7–11^, *Nannochloropsis oceanica*^12^, *Chlorella vulgaris*^13^, *Picochlorum celeri*^14^, *Euglena gracilis*^15^, *Coccomyxa *sp.^16^, *Tetraselmis *sp.^17^, and *Phaeodactylum tricornutum*^18^. These studies suggest that RNP-mediated genome editing is widely applicable to diverse microalgae. Based on this accumulated evidence, we considered that the RNP-based approach would also be effective in establishing genome editing in a non-model diatom.

The marine diatom *Fistulifera solaris* JPCC DA0580 is known for its exceptionally high oil productivity, with lipid content reaching up to 65% of the dry cell weight, primarily stored as triacylglycerol^19–21^. Recent genomic analysis has suggested that *F. solaris* possesses an allodiploid genome, containing two distinct sets of homoeologous chromosomes^21^. This genomic architecture presents both opportunities and challenges for genetic engineering, as successful gene knockout typically requires editing of both homoeologous copies to achieve a complete loss of function. Although metabolic engineering approaches have been applied to enhance oil productivity^22,23^, further improvement is essential for the commercial use of biofuel derived from *F. solaris*. Despite its proven utility in other microalgae, RNP-mediated genome editing has yet to be explored in *F. solaris*, and its applicability to this allodiploid species has not been validated.

In this study, we evaluated the applicability of RNP-mediated genome editing in *F. solaris* by targeting adenine phosphoribosyl transferase (*apt*) and diadinoxanthin de-epoxidase (*dde*) genes. Loss of APT activity confers resistance to the toxic adenine analog 2-fluoroadenine (2-FA) in several organisms, including plants and microalgae. The *apt* gene was selected because its disruption confers resistance to 2-fluoroadenine (2-FA), enabling efficient selection of edited cells. The *dde* genes encode a key enzyme in the xanthophyll cycle and were selected as model targets in this study because they are representative targets for improving the production of valuable compounds in diatoms. This work demonstrates RNP-mediated genome editing in a non-model oleaginous diatom *F. solaris.* These findings provide a valuable genetic tool for *F. solaris* and contribute to expanding the genetic engineering toolkit for diatoms.

## Experimental procedures

### Strain and culture conditions

The marine diatom *Fistulifera solaris* JPCC DA0580 was isolated from the mouths of the Sumiyo and Yakugachi Rivers in Amami-Ohsima, Kagoshima, Japan (28°15’N, 129°24’E)^24^. *F. solaris* JPCC DA0580 was maintained in half-strength Guillard’s f medium (f/2 medium) (75 mg NaNO_3_, 5 mg Na_2_HPO_4_·H_2_O, 0.5 µg vitamin B12, 0.5 µg biotin, 100 µg thiamine HCl, 30 mg Na_2_SiO_3_·9H_2_O, 4.36 mg Na_2_-EDTA, 3.15 mg FeCl_3_·6H_2_O, 10 µg CoSO_4_·5H_2_O, 22 µg ZnSO_4_·7H_2_O, 180 µg MnCl_2_·4H_2_O, 9.8 µg CuSO_4_·5H_2_O, and 6.3 µg Na_2_MoO_4_·2H_2_O)^25^ dissolved in artificial seawater (Marine art SF-1, Tomita Pharmaceutical Co., Ltd., Tokushima, Japan) containing 50 µg/ml ampicillin. The cells for transformation were cultured in flat flasks at 25 °C under continuous, cool-white fluorescent light at 130 µmol photons/m^2^/s in sterile air containing 2% CO_2_ at 0.8 vvm (volume of air per volume of medium per minute) with 10f medium. The 10f medium contains increased concentrations of NaNO_3_, NaH_2_PO_4_, NaSiO_3_, trace metals, and vitamins relative to the standard f/2 formulation, corresponding to approximately 20-fold higher nutrient concentrations than f/2 medium.

For the investigation of the sensitivity of *F. solaris* to 2-fluoroadenine (2-FA), f/2 agar medium supplemented with 2-FA at final concentrations of 0, 30, 50, and 75 µM was used. The *apt* gene knockout strain was cultured on f/2 medium supplemented with 2-FA at a final concentration of 75 µM.

### Design of gRNAs

Since *F. solaris* has an allodiploid genome^21^, gRNA target sites were selected from conserved regions shared between the two homoeologous copies. To identify the adenine phosphoribosyl transferase gene (*apt*) in *F. solaris*, a homology search against the whole genome sequence of *F. solaris* (GenBank accession no. GCA_002217885.1) was conducted (E-value ≤ 10^− 8^). The *apt* gene in *P. tricornutum* (XM_002183121) was used as the query. *P. tricornutum* was chosen as the reference species because its genome is well annotated, widely used as a model diatom, and phylogenetically close to *F. solaris*. BLAST searches putatively identified two apt homoeologous genes (gene ID: fso: g510, protein ID: GAX09716.1 and gene ID: fso: g10415, protein ID: GAX19605.1) in *F. solaris*. These two *apt* genes were selected as first targets for genome editing. To determine the *dde* in *F. solaris*, a homology search against the whole genome sequence of *F. solaris* was conducted (E-value ≤ 10^− 8^). The *dde* gene in *P. tricornutum* (XP_002178643) was used as the query. BLAST searches putatively identified two *dde* homoeologous genes (gene ID: fso: g1757, protein ID: GAX10961.1 and gene ID: fso: g3801, protein ID: GAX13004.1) in *F. solaris*. These were selected as the secondary targets for gene editing.

The Cas-Designer of the CRISPR RGEN Tool^26^ was used to select gRNA target sequences for gene knockout. The search was conducted under the following conditions: 1) a PAM sequence (5’-NGG-3’) must be located at the 3’ end of the target sequence; 2) the GC content of the target sequence must be between 20% and 80%; and 3) the out-of-frame score must be 66 or higher (this parameter was applied only for target selection in *dde* genes).

**Fig. 1:**
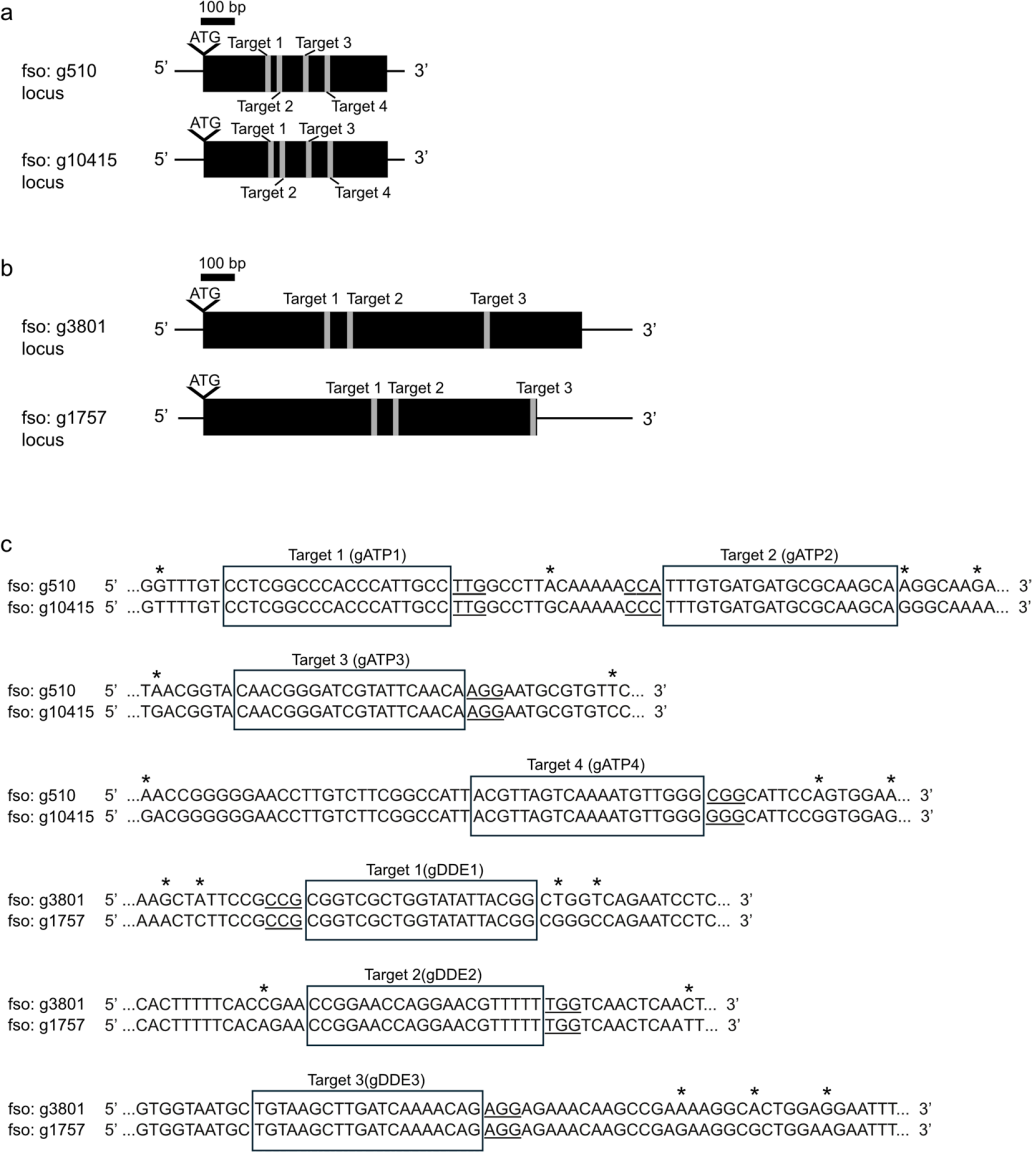
(a) Structures of the homoeologous *apt* genes (fso: g510 and fso: g10415) and positions of four gRNA target sites (Target 1–4). (b) Structures of the homoeologous *dde* genes (fso: g3801 and fso: g1757) and positions of three gRNA target sites (Target 1–3). Black boxes represent coding regions (exons), and vertical gray lines indicate target positions. ATG indicates the start codon. Target site positions are based on conserved sequences between the two homoeologous genes. (c) The alignment of target genes around gRNA target sites. The gRNA sequence is indicated by a boxed region and the PAM sequence is underlined. The asterisks indicate nucleotide differences between the two homoeologous genes.

For the *apt* gene, four gRNAs (gAPT1, gAPT2, gAPT3, and gAPT4) were designed, while for the *dde* gene, three gRNAs (gDDE1, gDDE2, and gDDE3) were designed (Fig. 1; Tables 1 and 2).

**Table 1:** The target sequences of each gRNA.

Target genes	Gene ID	gRNA names	Target sequences (5’−3’)	GC content (%)	Out-of-frame score
*apt*	fso: g510	gAPT1	CCTCGGCCCACCCATTGCCT**TGG**	70	77.9
	fso: g10415		CCTCGGCCCACCCATTGCCT**TGG**	70	73.2
	fso: g510	gAPT2	TGCTTGCGCATCATCACAAA**GGG**	45	53.5
	fso: g10415		TGCTTGCGCATCATCACAAA**TGG**	45	71.0
	fso: g510	gAPT3	CAACGGGATCGTATTCAACA**AGG**	45	56.6
	fso: g10415		CAACGGGATCGTATTCAACA**AGG**	45	49.2
	fso: g510	gAPT4	ACGTTAGTCAAAATGTTGGG**CGG**	40	69.0
	fso: g10415		ACGTTAGTCAAAATGTTGGG**GGG**	40	56.0

PAM sequences are in bold.

Out-of-frame score was calculated by using Cas-Designer.

**Table 2:** DNA sequences of gRNAs targeting *dde* genes.

Target genes	Gene ID	gRNA names	Target sequences (5’−3’)	GC contents (%)	Out-of-frame score
*dde*	fso: g1757	gDDE1	CCGTAATATACCAGCGACCG**CGG**	55	76.1
	fso: g3801		CCGTAATATACCAGCGACCG**CGG**	55	69.9
	fso: g1757	gDDE2	CCGGAACCAGGAACGTTTTT**TGG**	50	74.9
	fso: g3801		CCGGAACCAGGAACGTTTTT**TGG**	50	73.6
	fso: g1757	gDDE3	TGTAAGCTTGATCAAAACAG**AGG**	35	78.9
	fso: g3801		TGTAAGCTTGATCAAAACAG**AGG**	35	78.3

PAM sequences are in bold.

Out-of-frame score was calculated by using Cas-Designer.

### Preparation of CRISPR/Cas9 ribonucleoprotein complex (RNP)

crRNA and tracrRNA (each at 100 µM; IDT, Coralville, IA, USA) were mixed at equimolar concentrations in Nuclease-Free Duplex Buffer (IDT) to a final concentration of 10 µM each. The mixture was heated at 95 °C for 5 min and then cooled to room temperature to form gRNA. The resulting gRNA was then mixed with Alt-R S.p. Cas9 Nuclease V3 (62 µM; IDT) and phosphate-buffered saline (PBS) to obtain final concentrations of 1 µM gRNA and 1 µM Cas9 in a total volume of 100 µl, followed by incubation at room temperature for 15 min to assemble the RNP complex.

### In vitro cleavage assay of RNP complex

The in vitro cleavage assay was performed to verify the target-specific cleavage activity of each RNP. Genomic DNA was extracted from *F. solaris* using the Nucleospin Tissue XS kit (TaKaRa Bio, Japan). Target regions containing the *apt* and *dde* genes were amplified by PCR using gene-specific primer pairs (Table 3), yielding amplicons of approximately 1.2 kbp. Purified PCR products and RNPs were mixed with 10× Cas9 Nuclease Reaction Buffer (200 mM HEPES, 1 M NaCl, 50 mM MgCl₂, 1 mM EDTA, pH 6.5) at a molar ratio of 1:10 and incubated at 37 °C for 1 h. The reaction was terminated by adding proteinase K, followed by incubation at 56 °C for 10 min to inactivate Cas9. The reaction products were analyzed by agarose gel electrophoresis. Two controls were included: a no-enzyme control, in which purified PCR products were mixed with nuclease-free water, and a Cas9-only control, in which purified PCR products were incubated with Cas9 protein without gRNA.

**Table 3:** Primers used in this study.

Primer name	Sequences (5’ – 3’)	Use
g510 in vitro F3	GCGAAAGATGAATAGGTCTAATCTC	PCR amplification for in vitro assay, and sequencing of fso: g510
g510 in vitro R3	ATGACCCTACTCTCCATTGG	PCR amplification for in vitro assay of fso: g510
g10415 in vitro F3	GGCAAAGTTGTTAAAAAGGC	PCR amplification for in vitro assay, and sequencing of fso: g10415
g10415 in vitro R3	TGACCGTCTTATCCATTGG	PCR amplification for in vitro assay of fso: g10415
g510 seq R	GACTAATGACAAACAAACACCTG	PCR amplification of fso: g510
g10415_seq_R	AGAGTTAGTGCAGAGATGGGAC	PCR amplification of fso: g10415
g510 F	AGCAACAACAATAGCAAAGATG	2nd PCR amplification and sequencing of fso: g510
g510 R	GACTAATGACAAACAAACACCTG	2nd PCR amplification and sequencing of fso: g510
g10415 F	AACAACAACAATAGCAACGAC	2nd PCR amplification and sequencing of fso: g10415
g10415 R	GATCAATGAACATTTTGAGCTC	2nd PCR amplification and sequencing of fso: g10415
g1757 F1	GATACCACTCCCTGCCAGC	PCR amplification for in vitro assay, and sequencing of fso: g1757
g1757_R1	CGCCGTCGAAAGTAAGAAAC	PCR amplification for in vitro assay, and sequencing of fso: g1757
g1757_F2	ATCAAGTGCGGTGACATATTC	Sequencing of fso: g1757
g1757_R2	TAGTTCCTTTTCCAGTAACTCC	Sequencing of fso: g1757
g3801_F1	CAATATCGTCTCTTGCCTGTTCG	PCR amplification for in vitro assay, and sequencing of fso: g3801
g3801_R1	GGATTGCGCCGTAGGAATAAG	PCR amplification for in vitro assay, and sequencing of fso: g3801
g3801_F2	AATCAAGTGCGGTGATATATTTG	2nd PCR amplification and sequencing of fso: g3801

### Particle bombardment of Cas9 RNP complexes into *F. solaris*

Cells were collected at 48 h and spread onto 1% agar plates containing f/2 medium (5 × 10^7^ cells per plate). Particle bombardment of Cas9 RNP complexes was performed following the protocol of Russo et al. (2022)^27^, with minor modifications. Briefly, 0.6 μm gold particles were prepared as previously described^27^ and stored at −30 °C. Each 50 µl aliquot contained 3 mg of gold particles. On the day of use, aliquots were thawed at room temperature, washed twice with PBS, and resuspended in 50 µl of PBS. For each shot, 10 µl of gold particles were mixed with RNP solution described below, then distributed onto a macrocarrier and dried for approximately 2 h on a clean bench to prevent contamination. To evaluate the knockout efficiency of the *apt* gene, 4 µg of Cas9 RNP complexes (approximately 25 pmol in total; 8 µl), including 2 µg (approximately 12.5 pmol) each of gAPT1 and gAPT3 or gAPT2 and gAPT4, were introduced into *F. solaris* cells. For dde gene editing, a total of 16 µg of Cas9 RNP complexes (approximately 100 pmol; 8 µl) was used per experiment. This included 4 µg (approximately 25 pmol) each of gAPT1 and gAPT3 as a selection marker, combined with either gDDE1 and gDDE2 or gDDE1 and gDDE3, also at 4 µg (approximately 25 pmol) each. The RNP mixture was diluted with PBS to obtain the desired amount of RNP complexes. Delivery of RNPs was performed using a Biolistic PDS-1000/He Particle Delivery System. Vacuum was 27 inHg and burst pressure was 1,100 psi. After 4 days, all cells on the plates were suspended in f/2 medium and transferred to f/2 agar medium containing 75 µM 2-FA. Editing efficiencies were calculated as the number of 2-FA-resistant colonies obtained per 10^8^ input cells.

### Screening of edited clones

Genomic DNA was extracted from 2-FA-resistant mutants and wild-type strains using the Nucleospin Tissue XS kit. The extracted DNA was used as a template for PCR amplification of the *apt* or *dde* gene using the primers listed in Table 3. For clones in which amplification was not successful in the initial PCR, nested PCR was performed to amplify the respective gene regions. The resulting PCR products were purified, and mutations were identified by Sanger sequencing.

### Statistics

The sensitivity of *F. solaris* to 2-FA was evaluated using six biological replicates.

## Results

### Evaluation of the efficiency of *apt* gene knockout with RNP

**Fig. 2:**
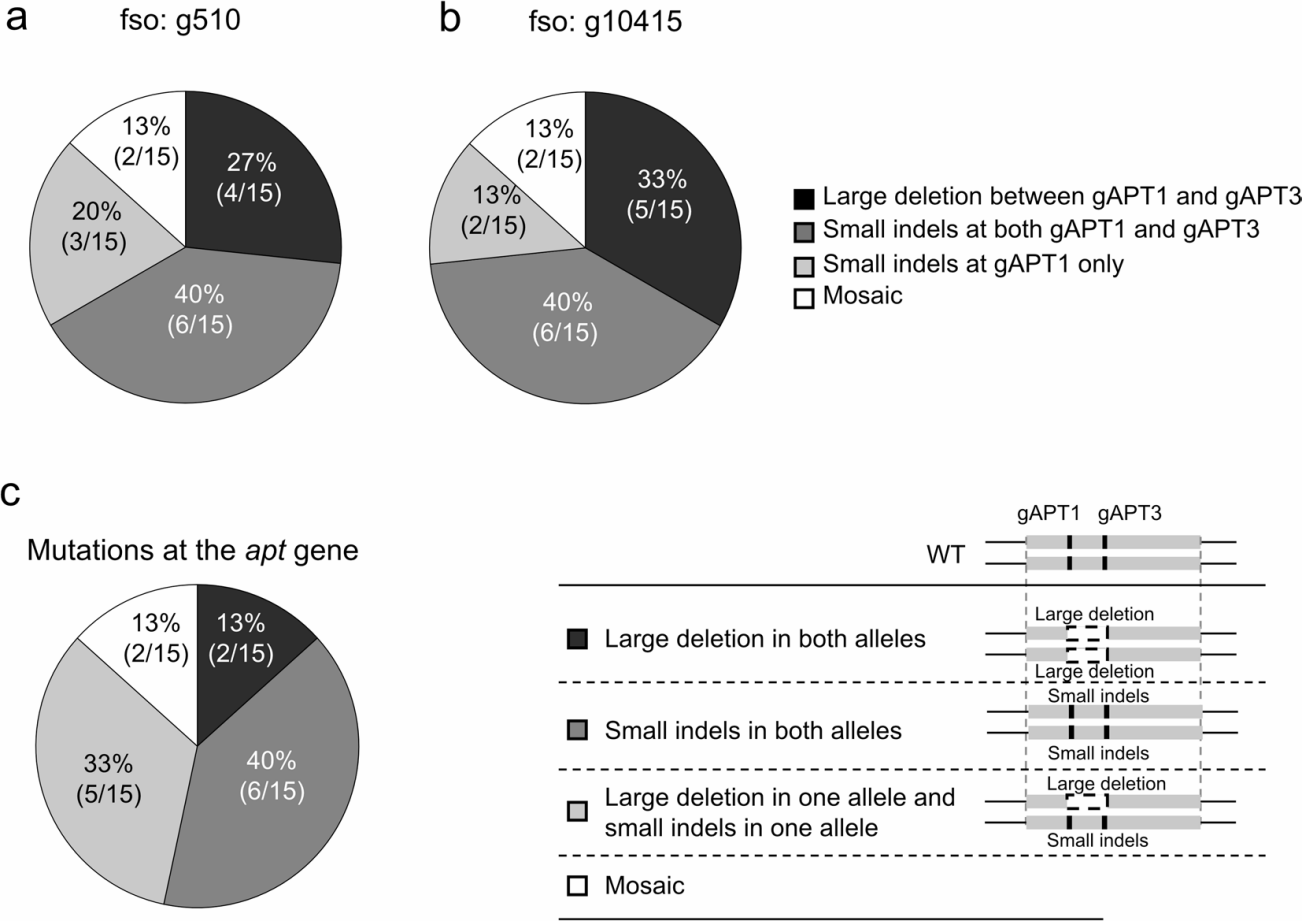
Frequency of mutagenesis of the *apt* genes in *F. solaris.* Frequency of colonies harboring mutation in (a) fso: g510 and (b) fso: g10415. Black sector: Large deletion between gAPT1 and gAPT3, Dark gray sector: Small indels at both gAPT1 and gAPT3, Light gray sector: Small indels at gAPT1 only, White sector: Mosaic. Mutations at only the gAPT3 target site were not observed. (c) Frequency of mutation types at *apt* genes. Black sector: large deletion in both alleles, Dark gray sector: Small insertion/deletion (indels) in both alleles, Light gray sector: large deletion in one allele and small indels in one allele, White sector: mosaic. Schematic representation shows the positions targeted by gATP1 and gAPT3. Black squares indicate the positions of gRNAs, and squares represent indels.

We demonstrate genome editing of *F. solaris* by direct introduction of ribonucleoprotein (RNP) complexes targeting the adenine phosphoribosyl transferase (*apt)* genes, which convert adenine and its analogs like 2-fluoroadenine (2-FA) into nucleotides. In wild-type cells, 2-FA is converted into toxic compounds by APT, whereas *apt*-knockout cells lack this activity and become resistant. This enables positive selection of edited cells using 2-FA^28^. To determine the appropriate selection conditions, the sensitivity of wild-type *F. solaris* to 2-FA was examined. Wild-type cells exhibited normal growth in the absence of 2-FA, whereas growth was strongly inhibited at concentrations above 75 µM, confirming that 2-FA is suitable for selection of *apt*-deficient mutants. Given the allodiploid nature of *F. solaris*, gRNAs were designed to target conserved sequences present in both homoeologous *apt* genes (fso: g510 and fso: g10415) to ensure simultaneous editing of both copies. Two gRNA pairs were designed: gAPT1–gAPT3 and gAPT2–gAPT4 (Fig. 1; Table 1). In an in vitro cleavage assay, gAPT2 exhibited the lowest cleavage efficiency against fso: g10415 among the tested gRNAs (Supplementary Fig. S1; uncropped images are provided in Supplementary Fig. S2). Therefore, gAPT1-gAPT3 was selected for knockout of *apt* genes based on their superior cutting efficiency. RNP complexes (4 µg) were delivered into *F. solaris* cells by particle bombardment. The transformation efficiency was calculated as the number of 2-FA-resistant colonies per total number of cells subjected to bombardment. After three weeks, 15 colonies resistant to 2-FA were obtained from approximately 5 × 10^7^ cells. All colonies grew in f/2 liquid medium containing 75 µM 2-FA, confirming the loss of APT function in both homoeologous genes. We searched for potential off-target sites of the *apt*-specific gRNAs in the *F. solaris* genome using Cas-OFFinder^29^ allowing an NGG PAM and up to two mismatches. No potential off-target loci were identified for any of the gRNAs targeting *apt* genes, indicating a low risk of off-target editing. Molecular analysis revealed mutations in both fso: g510 and fso: g10415. Figure 2 summarizes the mutation frequencies of the *apt* genes in *F. solaris*. The mutation types in the *apt* genes (fso: g510 and fso: g10415) are shown in Fig. 2a and b, respectively. Four distinct types of mutation patterns were identified through Sanger sequencing: large deletions between gRNA target sites, small indels at gAPT1 and/or gAPT3 sites, mosaic patterns where mutation types could not be clearly determined due to mixed sequences, and unedited sequences showing no detectable mutations. In this study, small insertions or deletions (indels) were defined as short insertions or deletions occurring around the predicted Cas9 cleavage site, typically within a few base pairs. Large deletions were defined as deletions covering the region between the two gRNA target sites (gAPT1 and gAPT3). Mixed Sanger chromatogram patterns are considered to result from colony mosaicism, which occurs when genome editing takes place after the initially transformed cell has already begun to divide, leading to the coexistence of wild-type and edited cells within the same colony^30,31^.

Detailed analysis of mutation patterns revealed consistent editing efficiency between the two homoeologous genes. For fso: g510, large deletions between gAPT1 and gAPT3 sites occurred in 27% of clones (4/15), small indels at both target sites in 40% (6/15), small indels at gAPT1 only in 20% (3/15), and mosaic patterns in 13% (2/15). Similarly, for fso: g10415, large deletions were observed in 33% of clones (5/15), small indels at both sites in 40% (6/15), small indels at gAPT1 only in 13% (2/15), and mosaic patterns in 13% (2/15). When considering biallelic mutations across both homoeologs, 13% of clones (2/15) showed large deletions in both alleles, 40% (6/15) had small indels in both alleles, and 33% (5/15) displayed mixed mutation types with large deletions in one allele and small indels in the other (Fig. 2c). In 13% (2/15) of clones, the mutation type could not be determined due to colony mosaicism. The overall biallelic mutation rate was 87% (13/15), demonstrating highly efficient simultaneous editing of both homoeologous genes. The editing efficiency obtained by RNP-mediated genome editing in this study (15 colonies/10^8^ cells) was markedly higher than that achieved using the previously reported vector-based Cas9/gRNA expression system for *F. solaris*^32^(0.3 colonies/10^8^ cells) (Table 4). In the previous study, genome editing was performed using a plasmid introduced by particle bombardment that expressed Cas9 and a gRNA targeting the same genomic site as gAPT1, allowing a direct comparison of the two methods.

**Table 4:** Comparison of genome editing efficiencies between the RNP-mediated and vector-based methods targeting the apt genes in *F. solaris*.

Method	Number of 2-FA-resistant colonies (colonies/10^8^ cells)	Number of positive clones to tested clones (clones/clones)	Proportion of clones with mutations at both homoeologous genes	Reference
RNP-mediated	15	15/15	15/15	This study
Vector-based	0.3	1/1	0/1	Previous study^32^

In this experiment, two RNPs targeting the *apt* genes (gAPT1 and gAPT3) were simultaneously introduced into *F. solaris*, and 2 µg of each RNP was delivered via particle bombardment. Data for the vector-based genome editing method are from our previous study^32^, in which *F. solaris* cells were transformed with a plasmid expressing Cas9 and a gRNA targeting the same genomic site as gAPT1 used in this study.

These results indicate that RNP-mediated editing is more efficient than vector-based approaches in this diatom species. We successfully achieved efficient genome editing in *F. solaris* using the RNP method.

### Multiplex RNP-mediated knockout of *dde* genes using *apt* gene selection

The *dde* genes (fso: g1757 and fso: g3801) encode diadinoxanthin de-epoxidase, a key enzyme in the xanthophyll cycle that is potential metabolic regulator involved in the biosynthesis of valuable compounds^33^^34^,. Disruption of *dde* genes is expected to enhance fucoxanthin accumulation in *F. solaris* by blocking the conversion of diadinoxanthin to diatoxanthin, thereby redirecting metabolic flux toward fucoxanthin biosynthesis. Similar to the *apt* gene targeting strategy, gRNAs were designed to target conserved sequences present in both homoeologous *dde* genes to ensure simultaneous editing of both copies in this allodiploid species. Two gRNA pairs were designed to knock out the *dde* genes: gDDE1–gDDE2 and gDDE1–gDDE3 (Table 2).

To establish a dual selection system, we employed *apt* gene knockout as a selectable marker alongside *dde* gene editing. A total of 16 µg of RNP was introduced, comprising 8 µg of gDDE1–gDDE2 RNP or gDDE1–gDDE3 RNP and 8 µg of gAPT1–gAPT3 RNP used as a selective marker. Particle bombardment was performed under identical conditions to those used for *apt* gene knockout. As a result, the number of 2-FA-resistant colonies obtained with gDDE1–gDDE2 RNP was higher than that obtained with gDDE1–gDDE3 RNP (Table 5). Based on this result, the editing efficiency was evaluated using the colonies obtained using gDDE1–gDDE2 RNP.

**Table 5:** Number of 2-FA-resistant colonies in obtained by multiplex RNP-mediated genome editing targeting both *apt* and *dde* genes.

gRNAs	Number of 2-FA-resistant colonies (colonies/10^8^ cells)
gDDE1, gDDE2	48.7
gDDE1, gDDE3	46

In this experiment, 4 µg of each RNP were introduced into *F. solaris.*

**Fig. 3:**
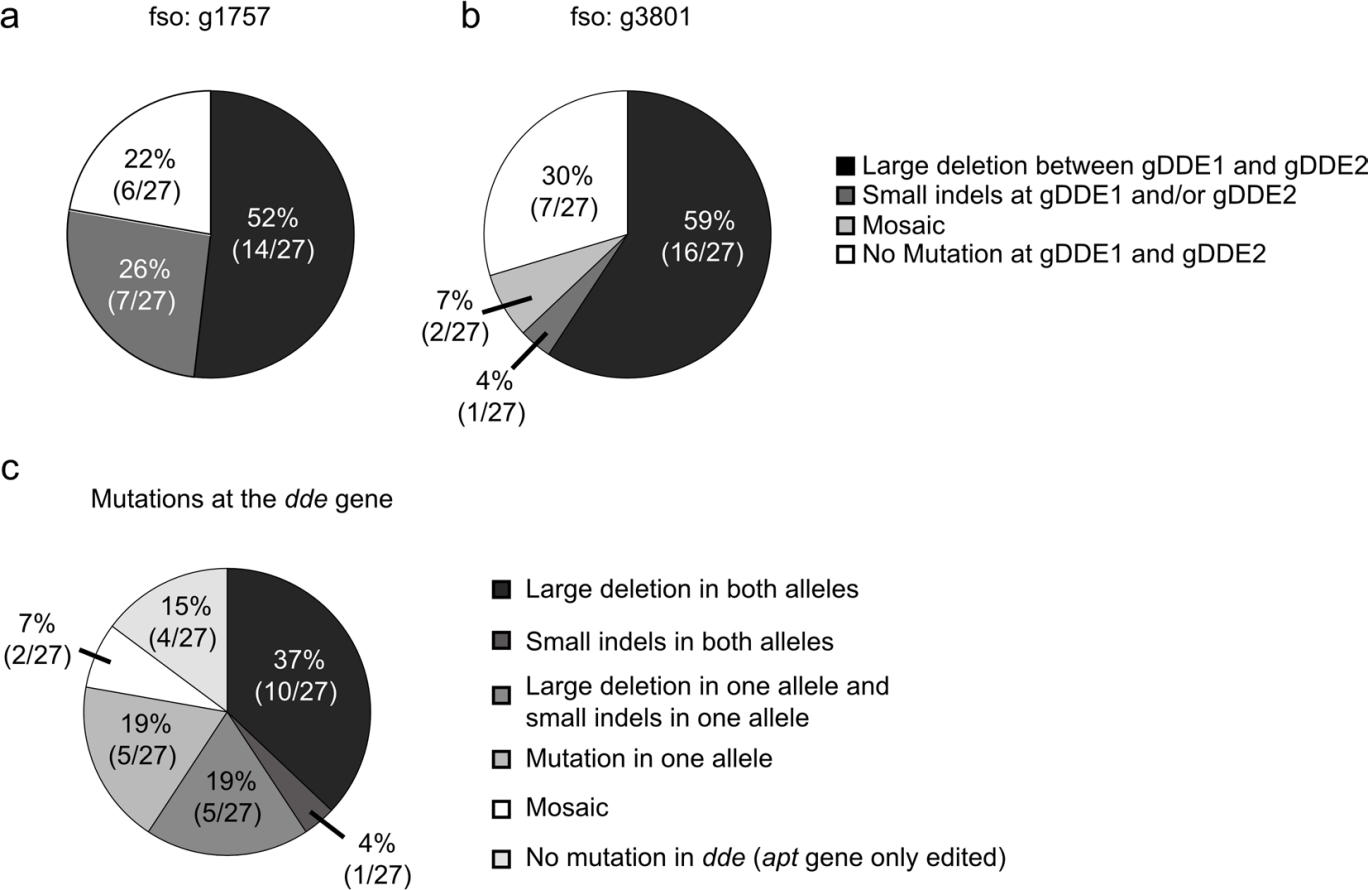
Frequency of mutagenesis in the *dde* genes with gDDE1 and gDDE2 in *F. solaris.* Frequency of 2-FA-resistant colonies harboring mutations in (a) fso: g1757 and (b) fso: g3801. Black sector: Large deletions, Dark gray sector: Small indels, Light gray sector: Mosaic, White sector: No mutation. (c) Frequency of mutation types in *dde* genes. Black sector: Mutation in both homoeologous genes, Dark gray sector: Mutation in one homoeologous gene, White sector: No mutation in *dde* genes and mutation in *apt* genes.

Twenty-seven clones were obtained through genome editing using gDDE1 and gDDE2 (the efficiency was 48.7 colonies/10^8^ cells in average). Mutations in the *apt* genes were detected in all 27 clones by Sanger sequencing. Subsequently, PCR amplification and Sanger sequencing were performed to analyze mutations in the *dde* genes. The mutation types in the *dde* genes (fso: g1757 and fso: g3801) are shown in Fig. 3a and b, respectively. Similar to the *apt* gene editing results, four types of outcomes were observed. Similarly, we searched for potential off-target sites of the *dde*-specific gRNAs in the *F. solaris* genome using Cas-OFFinder^29^ allowing an NGG PAM and up to two mismatches. No potential off-target loci were identified for any of the gRNAs targeting the dde genes, indicating a low risk of off-target editing.

Analysis of *dde* gene editing revealed varying degrees of knockout efficiency between the two homoeologous genes. For fso: g1757, mutations were detected in 78% of clones (21/27), with large deletions accounting for 52% (14/27), small indels for 26% (7/27), and mosaic patterns preventing clear determination in 22% (6/27). For fso: g3801, the editing efficiency was 63% (17/27), comprising 59% large deletions (16/27), 4% small indels (1/27), 7% mosaic patterns (2/27), and 30% unedited sequences (7/27). When considering biallelic editing of both homoeologous *dde* genes simultaneously, 59% of clones (16/27) showed mutations in both homoeologous genes (fso: g1757 and fso: g3801), while 19% (5/27) had mutations in only one homoeologous gene and 7% (2/27) had mosaic patterns. Four clones (15%) showed no detectable mutations in either *dde* gene, despite successful *apt* gene editing. As a result, the editing efficiency for at least one homoeologous *dde* gene was 85% (23/27) (Fig. 3c).

These results demonstrate the successful application of RNP-mediated gene editing for multiple target genes in *F. solaris*, providing a basis for further functional analysis in this oleaginous diatom. In this study, the analysis of *dde* mutants was limited to sequence-level characterization, and functional or metabolic consequences of *dde* disruption were not assessed.

## Discussion

In this study, we successfully established an RNP-mediated genome editing method in the oleaginous diatom *F. solaris*. Using the adenine phosphoribosyl transferase (*apt*) genes (fso: g510 and fso: g10415) as a selection marker and the diadinoxanthin de-epoxidase (*dde*) genes (fso: g1757 and fso: g3801) as potential metabolic targets, we demonstrated efficient genome editing at both target sites. Notably, this is the first report of successful RNP-mediated genome editing in *F. solaris*, expanding the toolkit for genetic manipulation of industrially relevant diatom species.

The allodiploid nature of *F. solaris* presents unique challenges for genome editing, as complete gene knockout typically requires successful editing of both homoeologous copies. In a previous study, *apt* gene knockout in *P. tricornutum* (a diploid species) was achieved via RNP-mediated genome editing using electroporation, resulting in 16 resistant clones, 93% of which had biallelic mutations^18^. Similarly, we targeted two homoeologous *apt* genes in *F. solaris* using particle bombardment and obtained 15 edited clones. Mutations were detected in 13 of edited clones, all of which exhibited biallelic mutations, representing an editing efficiency of 87%. These results demonstrate that RNP-mediated genome editing can also achieve high-efficiency biallelic mutations in *F. solaris*.

We also tested multiplex genome editing in *F. solaris* by co-targeting the *apt* and *dde* genes. This approach enabled simultaneous disruption of both loci. Although *dde* is associated with the xanthophyll cycle, this study did not evaluate the transcriptional or metabolic impact of *dde* knockout. Comprehensive metabolite and transcript analyses will be essential in future work. In a previous study on *P. tricornutum*, co-targeting of *apt* and *Aureochrome 1a* (*PtAureo1a*) genes resulted in mutations at the *PtAureo1a* locus in 65% of clones, with biallelic mutations confirmed in 52%^18^. In comparison, our co-targeting approach in *F. solaris* resulted in mutations in at least one of the homoeologous *dde* genes in 85% (23/27) of clones and biallelic mutations in 63% (17/27). These results demonstrate the successful application of RNP-mediated multiplex genome editing in *F. solaris*, achieving biallelic mutations exceeding 60% of co-targeted clones. In addition, the *dde* locus exhibited a higher proportion of large deletions than the apt locus. Previous studies using dual-gRNA CRISPR strategies have reported that large deletions occur more frequently when the distance between the two target sites is shorter^35^. In this study, the distance between gDDE1 and gDDE2 (approximately 90 bp) was shorter than that between gAPT1 and gAPT3 (approximately 130 bp). This shorter spacing likely facilitated the generation of large deletions at the *dde* locus.

Although this study successfully established RNP-mediated genome editing in *F. solaris* and achieved efficient disruption of the *apt* and *dde* genes, the functional consequences of *dde* knockout were not investigated. Because *dde* encodes a key enzyme in the xanthophyll cycle, comprehensive analysis of transcriptomic and metabolic changes will be required to validate the biological impact of its disruption. These functional evaluations are planned for future work.

The establishment of RNP-mediated genome editing in *F. solaris* provides a powerful and versatile platform for targeted genome editing and providing a foundation for future functional and metabolic studies. Diatoms and other microalgae are increasingly recognized for their potential in industrial applications. Nevertheless, only a limited number of species have been developed to a level that enables practical application. Many industrially promising strains remain non-model organisms with few available genetic tools, which constrains both strain development and functional gene characterization. Demonstrating that RNP-mediated genome editing is applicable to a non-model diatom, such as *F. solaris* represents a significant advance toward expanding genetic accessibility across diverse microalgal species. The ability to perform precise, DNA-free gene disruption in such organisms is expected to accelerate strain improvement and functional genomics, especially in species of significant industrial importance. Future applications could include knockout of genes involved in competing metabolic pathways, enhancement of lipid biosynthesis genes, or introduction of novel metabolic capabilities through targeted gene insertion. Moreover, this study highlights the applicability of RNP-mediated editing across diverse diatom species, including industrially relevant non-model species. The successful development of genome editing tools for *F. solaris* represents a significant step toward the rational design of diatom strains. Recent studies have demonstrated that CRISPR-based genome editing can effectively enhance the production of essential metabolites in several microalgae. In *P. tricornutum*, CRISPR knockout of zeaxanthin epoxidase stabilizes diatoxanthin accumulation, demonstrating successful carotenoid pathway engineering in diatoms^36^. CRISPR/Cas9 editing has also been applied in *Nannochloropsis *spp.^37^ to improve lipid productivity. Furthermore, RNP-mediated genome editing has been successfully used in *C. reinhardtii* for metabolic engineering of carotenoids^7^. These studies highlight the utility of the CRISPR/Cas9 system for modifying metabolic pathways in microalgae. Although this study focused on establishing an RNP-mediated genome editing platform in *F. solaris*, the method developed here provides a foundation for future applications in functional genomics and metabolic engineering in this industrially relevant diatom.

## Conclusion

In this study, we established a highly efficient RNP-mediated genome editing technique in the oleaginous diatom *F. solaris*. This study represents a successful application of RNP-mediated genome editing in this allodiploid species, achieving 87% biallelic knockout efficiency for *apt* genes and 63% for *dde* genes. The editing efficiency was comparable to that reported in the model diatom *P. tricornutum*, demonstrating that RNP-mediated genome editing is also applicable to the non-model species *F. solaris*. We also successfully demonstrated multiplex genome editing by simultaneously targeting the *apt* and *dde* genes. This method serves as a powerful tool for metabolic engineering aimed at enhancing biomass and oil productivity in *F. solaris* and further contributes to the development of genome editing techniques in other non-model diatom species.

## Electronic Supplementary Material

Below is the link to the electronic supplementary material.

Supplementary Material 1

## Data Availability

No datasets were generated or analysed during the current study.

## References

[1] Bwapwa, J. K., Anandraj, A. & Trois, C. Possibilities for conversion of microalgae oil into aviation fuel: A review. *Renew. Sustain. Energy Rev.* **80**, 1345–1354. 10.1016/j.rser.2017.05.224 (2017).

[2] Wang, M., Ye, X., Bi, H. & Shen, Z. Microalgae biofuels: illuminating the path to a sustainable future amidst challenges and opportunities. *Biotechnol. Biofuels Bioprod.* **17**, 10. 10.1186/s13068-024-02461-0 (2024). 38254224 10.1186/s13068-024-02461-0 PMC10804497

[3] Sharma, P. K., Saharia, M., Srivstava, R., Kumar, S. & Sahoo, L. Tailoring Microalgae for Efficient Biofuel Production. *Front. Mar. Sci.* 5–2018. 10.3389/fmars.2018.00382 (2018).

[4] Barka, F. et al. Identification of a triacylglycerol lipase in the diatom Phaeodactylum tricornutum. *Biochim. et Biophys. Acta (BBA) - Mol. Cell. Biology Lipids*. **1861**, 239–248. 10.1016/j.bbalip.2015.12.023 (2016). 10.1016/j.bbalip.2015.12.023 26747649

[5] Jeon, S. et al. Current status and perspectives of genome editing technology for microalgae. *Biotechnol. Biofuels*. **10**, 267. 10.1186/s13068-017-0957-z (2017). 29163669 10.1186/s13068-017-0957-z PMC5686953

[6] Lattanzi, A. et al. Optimization of CRISPR/Cas9 Delivery to Human Hematopoietic Stem and Progenitor Cells for Therapeutic Genomic Rearrangements. *Mol. Ther.* **27**, 137–150. 10.1016/j.ymthe.2018.10.008 (2019). 30424953 10.1016/j.ymthe.2018.10.008 PMC6318785

[7] Baek, K. et al. DNA-free two-gene knockout in *Chlamydomonas reinhardtii* via CRISPR-Cas9 ribonucleoproteins. *Sci. Rep.* **6**, 30620. 10.1038/srep30620 (2016). 27466170 10.1038/srep30620 PMC4964356

[8] Dhokane, D., Bhadra, B. & Dasgupta, S. CRISPR based targeted genome editing of *Chlamydomonas reinhardtii* using programmed Cas9-gRNA ribonucleoprotein. *Mol. Biol. Rep.* **47**, 8747–8755. 10.1007/s11033-020-05922-5 (2020). 33074412 10.1007/s11033-020-05922-5

[9] Ferenczi, A., Pyott, D. E., Xipnitou, A. & Molnar, A. Efficient targeted DNA editing and replacement in *Chlamydomonas reinhardtii* using Cpf1 ribonucleoproteins and single-stranded DNA. *Proceedings of the National Academy of Sciences* 114, 13567–13572 (2017). 10.1073/pnas.1710597114 10.1073/pnas.1710597114 PMC5754772 29208717

[10] Nievergelt, A. P., Diener, D. R., Bogdanova, A., Brown, T. & Pigino, G. Efficient precision editing of endogenous *Chlamydomonas reinhardtii* genes with CRISPR-Cas. *Cell. Rep. Methods*. **3**10.1016/j.crmeth.2023.100562 (2023). 10.1016/j.crmeth.2023.100562 PMC10475843 37671018

[11] Picariello, T. et al. TIM, a targeted insertional mutagenesis method utilizing CRISPR/Cas9 in *Chlamydomonas reinhardtii*. *PLOS ONE*. **15**, e0232594. 10.1371/journal.pone.0232594 (2020). 32401787 10.1371/journal.pone.0232594 PMC7219734

[12] Naduthodi, M. I. S. et al. CRISPR-Cas ribonucleoprotein mediated homology-directed repair for efficient targeted genome editing in microalgae *Nannochloropsis oceanica* IMET1. *Biotechnol. Biofuels*. **12**, 66. 10.1186/s13068-019-1401-3 (2019). 30962821 10.1186/s13068-019-1401-3 PMC6432748

[13] Kim, J., Chang, K. S., Lee, S. & Jin, E. Establishment of a Genome Editing Tool Using CRISPR-Cas9 in *Chlorella vulgaris* UTEX395. *Int. J. Mol. Sci.* **22**10.3390/ijms22020480 (2021). 10.3390/ijms22020480 PMC7825080 33418923

[14] Krishnan, A., Cano, M., Burch, T. A., Weissman, J. C. & Posewitz, M. C. Genome editing using Cas9-RNA ribonucleoprotein complexes in the high-productivity marine alga *Picochlorum celeri*. *Algal Res.* **49**, 101944. 10.1016/j.algal.2020.101944 (2020).

[15] Nomura, T. et al. Highly efficient transgene-free targeted mutagenesis and single-stranded oligodeoxynucleotide-mediated precise knock-in in the industrial microalga *Euglena gracilis* using Cas9 ribonucleoproteins. *Plant Biotechnol. J.* **17**, 2032–2034. 10.1111/pbi.13174 (2019). 31131534 10.1111/pbi.13174 PMC6790356

[16] Yoshimitsu, Y., Abe, J. & Harayama, S. Cas9-guide RNA ribonucleoprotein-induced genome editing in the industrial green alga *Coccomyxa* sp. strain KJ. *Biotechnol. Biofuels*. **11**, 326. 10.1186/s13068-018-1327-1 (2018). 30555532 10.1186/s13068-018-1327-1 PMC6287348

[17] Chang, K. S. et al. Enhanced lipid productivity in AGP knockout marine microalga *Tetraselmis* sp. using a DNA-free CRISPR-Cas9 RNP method. *Bioresour. Technol.* **303**, 122932. 10.1016/j.biortech.2020.122932 (2020). 32058903 10.1016/j.biortech.2020.122932

[18] Serif, M. et al. One-step generation of multiple gene knock-outs in the diatom *Phaeodactylum tricornutum* by DNA-free genome editing. *Nat. Commun.* **9**, 3924. 10.1038/s41467-018-06378-9 (2018). 30254261 10.1038/s41467-018-06378-9 PMC6156588

[19] Liang, Y. et al. Dynamic oil body generation in the marine oleaginous diatom *Fistulifera solaris* in response to nutrient limitation as revealed by morphological and lipidomic analysis. *Algal Res.* **12**, 359–367. 10.1016/j.algal.2015.09.017 (2015).

[20] Matsumoto, M. et al. Marine diatom, *Navicula* sp. strain JPCC DA0580 and marine green alga, *Chlorella* sp. strain NKG400014 as potential sources for biodiesel production. *Appl. Biochem. Biotechnol.* **161**, 483–490. 10.1007/s12010-009-8766-x (2010). 19756412 10.1007/s12010-009-8766-x

[21] Tanaka, T. et al. Oil accumulation by the oleaginous diatom *Fistulifera solaris* as revealed by the genome and transcriptome. *Plant. Cell.* **27**, 162–176. 10.1105/tpc.114.135194 (2015). 25634988 10.1105/tpc.114.135194 PMC4330590

[22] Osada, K. et al. Enhanced NADPH production in the pentose phosphate pathway accelerates lipid accumulation in the oleaginous diatom *Fistulifera solaris*. *Algal Res.* **23**, 126–134. 10.1016/j.algal.2017.01.015 (2017).

[23] Maeda, Y. et al. Assessment on the oil accumulation by knockdown of triacylglycerol lipase in the oleaginous diatom *Fistulifera solaris*. *Sci. Rep.* **11**, 20905. 10.1038/s41598-021-00453-w (2021). 34686744 10.1038/s41598-021-00453-w PMC8536745

[24] Matsumoto, M. et al. Morphological and molecular phylogenetic analysis of the high triglyceride-producing marine diatom, *Fistulifera solaris* sp. nov. (Bacillariophyceae). *Phycological Res.* **62**, 257–268. 10.1111/pre.12066 (2014).

[25] Guillard, R. R. & Ryther, J. H. Studies of marine planktonic diatoms. I. *Cyclotella nana* Hustedt, and *Detonula confervacea* (cleve) Gran. *Can. J. Microbiol.* **8**, 229–239. 10.1139/m62-029 (1962). 13902807 10.1139/m62-029

[26] Park, J., Bae, S. & Kim, J. S. Cas-Designer: a web-based tool for choice of CRISPR-Cas9 target sites. *Bioinformatics* **31**, 4014–4016. 10.1093/bioinformatics/btv537 (2015). 26358729 10.1093/bioinformatics/btv537

[27] Russo, M. T., Santin, A., Rogato, A. & Ferrante, M. I. Optimized Proteolistic Protocol for the Delivery of the Cas9 Protein in *Phaeodactylum tricornutum*. *Methods Mol. Biol.* **2498**, 327–336. 10.1007/978-1-0716-2313-8_18 (2022). 35727554 10.1007/978-1-0716-2313-8_18

[28] Gaillard, C., Moffatt, B. A., Blacker, M. & Laloue, M. Male sterility associated with APRT deficiency in *Arabidopsis thaliana* results from a mutation in the gene APT1. *Mol. Gen. Genet. MGG*. **257**, 348–353. 10.1007/s004380050656 (1998). 9520269 10.1007/s004380050656

[29] Bae, S., Park, J. & Kim, J. S. Cas-OFFinder: a fast and versatile algorithm that searches for potential off-target sites of Cas9 RNA-guided endonucleases. *Bioinformatics* **30**, 1473–1475. 10.1093/bioinformatics/btu048 (2014). 24463181 10.1093/bioinformatics/btu048 PMC4016707

[30] Daboussi, F. et al. Genome engineering empowers the diatom *Phaeodactylum tricornutum* for biotechnology. *Nat. Commun.* **5**, 3831. 10.1038/ncomms4831 (2014). 24871200 10.1038/ncomms4831

[31] Hopes, A., Nekrasov, V., Kamoun, S. & Mock, T. Editing of the urease gene by CRISPR-Cas in the diatom *Thalassiosira pseudonana*. *Plant. Methods*. **12**, 49. 10.1186/s13007-016-0148-0 (2016). 27904648 10.1186/s13007-016-0148-0 PMC5121945

[32] Murata, S. et al. Establishment of genome editing techniques in the marine oleaginous diatom *Fistulifera solaris* for improved oil accumulation. *J. Biosci. Bioeng.*10.1016/j.jbiosc.2025.07.008 (2025). 40803956 10.1016/j.jbiosc.2025.07.008

[33] Li, C., Pan, Y., Yin, W., Liu, J. & Hu, H. A key gene, violaxanthin de-epoxidase-like 1, enhances fucoxanthin accumulation in *Phaeodactylum tricornutum*. *Biotechnol. Biofuels Bioprod.* **17**, 49. 10.1186/s13068-024-02496-3 (2024). 38566219 10.1186/s13068-024-02496-3 PMC10986045

[34] Bai, Y. et al. Green diatom mutants reveal an intricate biosynthetic pathway of fucoxanthin. *Proc. Natl. Acad. Sci.* **119** (e2203708119). 10.1073/pnas.2203708119 (2022). 10.1073/pnas.2203708119 PMC9499517 36095219

[35] Cui, X. et al. An optimised CRISPR/Cas9 protocol to create targeted mutations in homoeologous genes and an efficient genotyping protocol to identify edited events in wheat. *Plant. Methods*. **15**, 119. 10.1186/s13007-019-0500-2 (2019). 31673276 10.1186/s13007-019-0500-2 PMC6814032

[36] Græsholt, C. et al. Zeaxanthin epoxidase 3 Knockout Mutants of the Model Diatom *Phaeodactylum tricornutum* Enable Commercial Production of the Bioactive Carotenoid Diatoxanthin. *Mar. Drugs*. **22**10.3390/md22040185 (2024). 10.3390/md22040185 PMC11051370 38667802

[37] Ajjawi, I. et al. Lipid production in *Nannochloropsis gaditana* is doubled by decreasing expression of a single transcriptional regulator. *Nat. Biotechnol.* **35**, 647–652. 10.1038/nbt.3865 (2017). 28628130 10.1038/nbt.3865

