# Supplementary material for: Highly efficient genome editing using CRISPR/Cas9 ribonucleoprotein in the marine oleaginous diatom Fistulifera solaris
Source: Sci Rep. 2026 Apr 24;16:18912. doi: 10.1038/s41598-026-49816-1 (PMC13276009; doi:10.1038/s41598-026-49816-1)
Supplement: Supplementary file 1 — Supplementary Material 1 [file 41598_2026_49816_MOESM1_ESM.pdf]

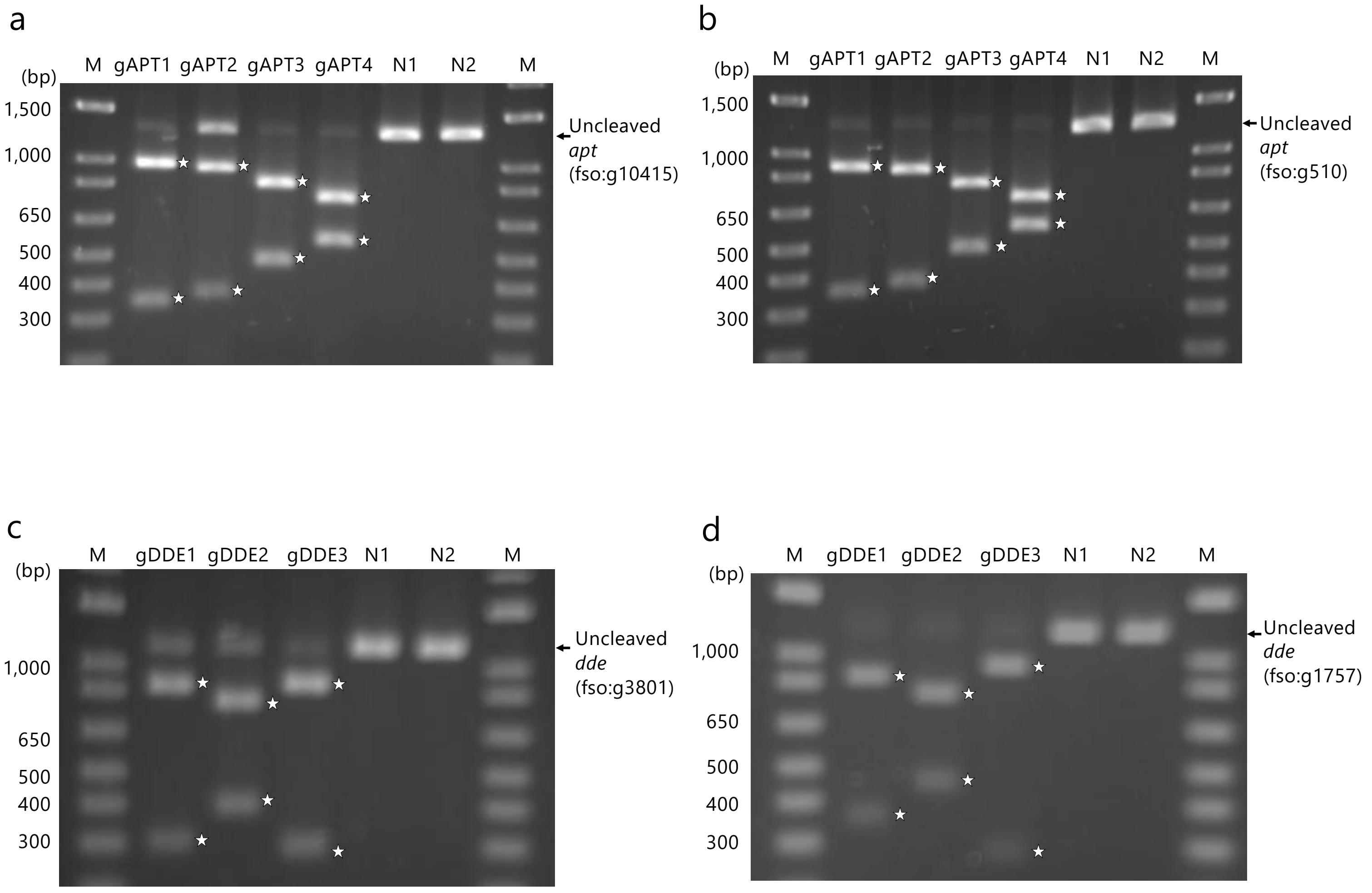

Fig. S1 *In vitro* cleavage assay of gRNAs targeting *apt* genes: (a) fso:g10415, (b) fso:g510, and *dde* genes: (c) fso:g1757, (d) g3801.

PCR amplicons containing each target site were incubated with Cas9–gRNA RNPs. M: marker, gAPT1–4: the cleavage fragments produced by each apt– targeting gRNA with Cas9, gDDE1–3: the cleavage fragments produced by each dde– targeting gRNA with Cas9, N1: the no-enzyme control (PCR product + nuclease-free water), N2: the Cas9-only control (PCR product + Cas9 without gRNA). Asterisks indicate cleaved fragments.

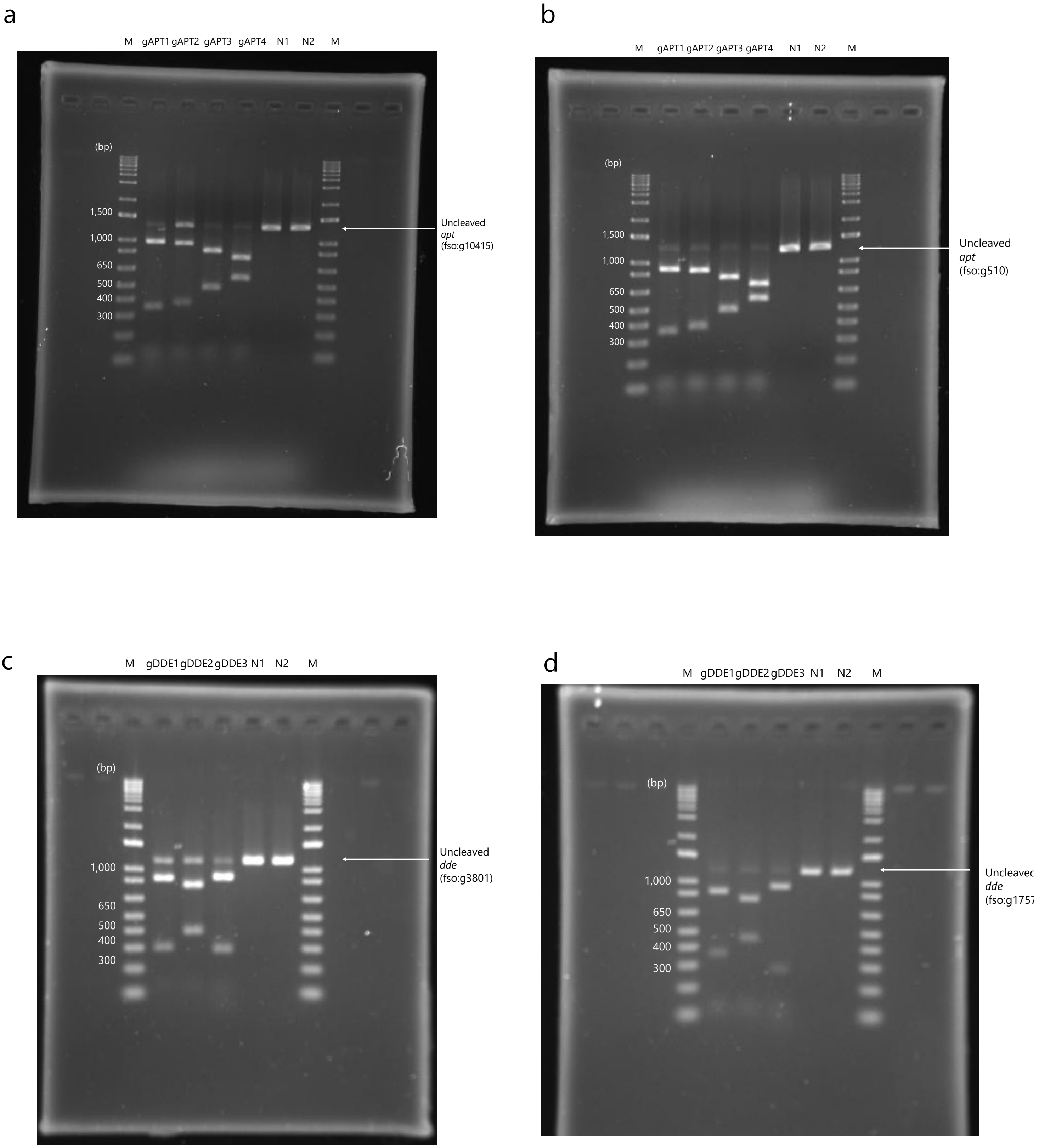

Fig. S2 Original uncropped gel images corresponding to Fig. S1.

(a–d) Full-length gel images with visible gel edges corresponding to Fig. S1 (a)–(d), respectively: (a) fso:g10415, (b) fso:g510, (c) fso:g1757, and (d) fso:g3801.

PCR amplicons containing each target site were incubated with Cas9–gRNA RNPs. M: marker, gAPT1–4: the cleavage fragments produced by each apt– targeting gRNA with Cas9, gDDE1–3: the cleavage fragments produced by each dde– targeting gRNA with Cas9, N1: the no-enzyme control (PCR product + nuclease-free water), N2: the Cas9-only control (PCR product + Cas9 without gRNA).
